# Clinical application of high frequency jet ventilation in stereotactic liver ablations – a methodological study

**DOI:** 10.12688/f1000research.14873.2

**Published:** 2018-09-10

**Authors:** Karolina Galmén, Jacob Freedman, Grzegorz Toporek, Waldemar Goździk, Piotr Harbut

**Affiliations:** 1 Department of Anaesthesiology and Intensive Therapy, Danderyd University Hospital, Stockholm, Sweden; 2 Department of Surgery and Urology, Danderyd University Hospital, Stockholm, Sweden; 3 ARTORG Center for Biomedical Engineering, University of Bern, Bern, Switzerland; 4 Department of Anaesthesiology and Intensive Therapy, Wrocław Medical University, Wrocław, Poland

**Keywords:** High frequency jet ventilation, Liver ablation, Stereotactic surgery

## Abstract

**Background: **Computer-assisted navigation during thermal ablation of liver tumours, may help to correct needle placement and improve ablation efficacy in percutaneous, laparoscopic and open interventions. The potential advantage of using high frequency jet-ventilation technique (HFJV) during the procedure is by minimising the amplitude of respiration-related upper-abdominal organs movements. The aim of this clinical methodological trial was to establish whether HFJV would give less ventilatory induced liver movements than conventional ventilation, during stereotactic navigated ablation of liver metastases under open surgery.

**Methods: **Five consecutive patients scheduled for elective, open liver ablation under general propofol and remifentanil anaesthesia were included in the study protocol. During the stereotactic targeting of the tumours, HFJV was chosen for intraoperative lung ventilation. For tracking of liver movement, a rigid marker shield was placed on the liver surface and tracked with an optical position measurement system. A 4D position of the marker shield was measured for HFJV and conventional tidal volume lung ventilation (TV). At each time point the magnitude of liver displacement was calculated as an Euclidean distance between translational component of the marker shield's 3D position and previously estimated centroid of the translational motion.

**Results:** The mean Euclidean liver displacement was 0.80 (0.10) mm for HFJV and 2,90 (1.03) mm for TV with maximum displacement going as far as 12 mm on standard ventilation (p=0.0001).

**Conclusion: **HFJV is a valuable lung ventilation method for patients undergoing stereotactic surgical procedures in general anaesthesia when reduction of organ displacement is crucial.

## Introduction

Thermal ablation of primary and secondary liver tumours is a potentially curative treatment, and an alternative for patients not eligible for surgical resection due to severe comorbidity or underlying liver disease. Its efficacy has been proven for tumours smaller than 30mm in diameter, especially in treatment of hepatocellular carcinomas ^1^. Adequate imaging of the tumour and precise guidance of the ablation device are crucial for accurate local ablative treatment. Accurate targeting is essential for an effective treatment, reducing the risk for local recurrence and need of retreatment ^1, 2^.

Recent developments in image guidance systems, with robotic and computer-assisted navigation, may help correct needle placement and improved ablation efficacy. Needle navigation and placement is based on pre-interventional imaging. Early phantom and clinical experiences with navigation systems suggest good procedural accuracy, reduced procedure time and reduced patient radiation exposure compared to freehand techniques ^3^.

The high frequency jet-ventilation technique (HFJV) was developed in the seventies by Klain and Smith, and mostly applied in the field of ear-nose-and-throat (ENT) surgery. It does not rely on conventional tidal volumes but uses a high frequency forced gas move ^4^. The potential advantage of using HFJV in abdominal surgery is to minimise the amplitude of respiration-related upper-abdominal organs compared to conventional tidal volume lung ventilation (TV) ^5– 7^.

The aim of this clinical methodological trial was to measure the liver movements during open surgery under general anaesthesia and compare HFJV with conventional ventilation.

## Methods

Five consecutive patients who were scheduled for elective, open liver ablations were included in the clinical protocol.

### Anaesthesia management protocol

General anaesthesia was induced and maintained by total intravenous technique (TIVA) with target controlled infusion (TCI - Alaris, PK CareFusion, Sarl, Switzerland) of propofol 2–6µg/ml according to Marsh pharmacokinetic model (Propofol Sandoz®, Sandoz, Copenhagen, Denmark) and remifentanil 2-10ng/ml according to Minto pharmacokinetic model (Ultiva®, GlaxoSmithKline,Solna, Sweden) with muscle relaxation achieved by rocuronium 0,6 mg/kg during induction of anaesthesia, followed by incremental doses of 0,15mg/kg during surgery (Rocuronium, Fresenius Kabi, Uppsala, Sweden).

### Lung ventilation and surgical precision evaluation

Endotracheal intubation with a conventional endotracheal (ET) tube was performed at the induction of anaesthesia, followed by the initiation of conventional lung ventilation with pressure control/volume guarantee ventilation (PCV/VG - Aisys Carestation, GE Healthcare, Helsinki, Finland) as well as a lung-protective regime to achieve normo-ventilatory status. Tidal volumes have been calculated after the reduced body weight with 6–7ml/kg target and fixed 5cmH20 positive end-expiratory pressure (PEEP). Laparotomy was performed with a right subcostal incision. A HFJV cannula (LaserJet Catheter, Acutronic Medical Systems AG, Hirzel, Switzerland) was inserted endotracheally with the tip at the end of the ET-tube. HFJV (Monsoon HFJV ventilator, Acutronic Medical Systems, AG, 8816 Hirzel, Switzerland) was then initiated and continued during the liver ablation procedure. HFJV driving pressure (DP) was adjusted downwards, beginning at 1.8 bar, until satisfactory operation-field conditions were reached in accordance with the operating surgeon’s assessment.

### Carbon dioxide control

In the first phase, after the induction of anaesthesia, end-tidal carbon dioxide tension (EtCO2) was continuously monitored through the use of classical side-stream capnography towards the normocapnic state. During the HFJV phase, sequential measurements were taken with 10 minute intervals (Integrated Monsoon ventilator etCO2 module). After the termination of the last tumour ablation and the completion of liver movement measurement, the conventional lung ventilation was restored. Lastly, the etCO2 measurement was repeated following the same method as the one used at the start of the procedure. Cut-off values for discontinuation of HFJV was either etCO2 rise over 10 kPa, or oxygen de-saturation under 90%. With etCO2 exceeding 8 kPa, the DP down-regulation has been stopped and instead increased by 0.1 bar increments every 5 minutes until the target etCO2 was reached.

### Surgical imaging and liver motion measurements

Patients were selected at the regional liver multidisciplinary team conference and were regarded as unresectable due to multiple metastases involving too many liver segments, but numbered less than twenty and none larger than thirty millimeters in diameter ^8^. Multiple ablations were then performed using an intraoperative ultrasound and a stereotactic targeting device, CAS-one (Cascination AG, Bern, Switzerland) where a previously acquired computed tomography scan was merged with previous scans in cases of vanished lesions, and a 3D model of the liver reconstructed by MeVIS medical solutions AG (Bremen, Germany) was used as a surgical map with optical navigation of ablation antennae, as previously described ^8^. For tracking of liver movement, a rigid marker shield with a set of retroreflective marker spheres was placed on the liver surface in the vicinity of the lower border of segment 4b and tracked with an optical position measurement system (Polaris Vicra, NDI, Canada) incorporated into the CAS-One system which was positioned in the vicinity of the operative field thus providing a constant line of sight. A 4D position of the marker shield was measured for approximately 2–3 minutes for HFJV and conventional ventilation.

At each time point *t*, the magnitude of liver displacement *d*, was calculated as an Euclidean distance between translational component $\vec{p}$ of the marker shield’s 3D position, and previously estimated centroid of the translational motion $\underline{c}$, i.e. an average translational position of the marker, as listed in the equation below:

*d*_*t*_ = ||⬚ *c* − ${\vec{p}}_{t}$||

All displacement errors *d* were described quantitatively using mean (µ) and standard deviation (σ) as well as a maximum error value. Statistically significant differences were tested with the two-tailed, nonparametric, unpaired t-test, where p < 0.05 was defined as statistically significant.

## Results

Patient demographics, medical status and extent of surgery is presented in Table 1.

**Table 1.** Patient demographics, medical status and extent of surgery.

Patient ID	Sex	Age (years)	ASA	Weight (kg)	Height (cm)	BMI	No lesions
1	m	74	3	78	178	25	4
2	m	58	4	99	178	31	8
3	m	76	3	82	181	25	2
4	m	82	3	77	185	23	9
5	f	48	3	73	163	27	30

*Patient characteristics. ASA=American Society of Anesthesiologists (ASA) Physical Status, BMI=Body Mass Index (kg/m ^2^)*

Ventilator settings and readings are shown in in Table 2. The following parameters have been registered: end tidal CO _2_ concentrations before and after HFJV phase, respiratory pressures on conventional tidal volume ventilation before and after HFJV, peak inspiratory pressure and mean airway before and after HFJV, mean airway pressure, dynamic lung compliance both before and after HFJV phase as well as tidal volumes on conventional lung ventilation, at liver displacement measurement point. HFJV ventilator settings: respiratory frequency and target driving pressure as well as the measured respiratory parameters: peak inspiratory pressure and mean airway pressure as well as maximum end tidal carbon dioxide tension on HFJV.

**Table 2.** Ventilator settings and readings.

Patient ID	1	2	3	4	5
etCO _2_ pre jet (%)	5.5	4.4	4.6	5.1	4.3
etCO _2_ post jet (%)	6	5.2	4.4	7	4.7
PeakP pre jet (cm. H _2_O)	14	16	15	13	14
MaP pre jet (cm. H _2_O)	8	9	9	10	8
Compliance pre jet (ml/cm. H _2_O)	64	79	54	73	53
TV post jet (ml)	507	607	585	507	465
Compliance post jet (ml/cm. H _2_O)	55	66	33	67	52
Time on jet (min)	40	97	45	70	165
FQ on jet (cpm)	220	220	220	220	220
T-DP on jet (bar)	1.2	1.5	1.1	1.2	1.2
PIP on jet (mbar)	7	7	6	5	7
MaP on jet (mbar)	4	5	4	3	4
Max etCO _2_ on jet (kPa)	6.5	8.3	4.7	6.7	4.8

*End tidal CO _2_ (etCO _2_)registered before and after High frequency jet ventilation (HFJV) phase (etCO _2_ - pre jet and post jet), Respiratory pressures on conventional tidal volume ventilation before and after HFJV phase, expressed in cm.H _2_O: peak inspiratory pressure (PeakP) pre jet, mean airway pressure (MaP) pre jet, dynamic lung compliance both before and after HFJV phase (Compliance pre and post jet) as well as tidal volumes on conventional lung ventilation, at liver displacement measurement point (TV post jet). HFJV ventilator settings: respiratory frequency (FQ on jet) and target driving pressure (T-DP on jet) as well as the measured respiratory parameters: peak inspiratory pressure (PIP) and mean airway pressure (MaP) and maximum end tidal carbon dioxide tension during HFJV phase (Max etCO _2_ on jet).*

In one case (patient number 2) an increase in DP was needed, because the etCO2 rose to 8.3 kPa and the optimal value was set on to 1.5 bar where in other four cases it was set to 1.1–1.2 bar. One patient (number 5) turned out to have more than 20 metastases at time of surgery, but not on the imageing where the treatment was allocated. Rather than to close up, a new evaluation was done during surgery using intraoperative ultrasound and it was found that ablations could be done leaving an adequate volume of functioning liver parenchyma, why this was the course taken.

**Figure 1.**
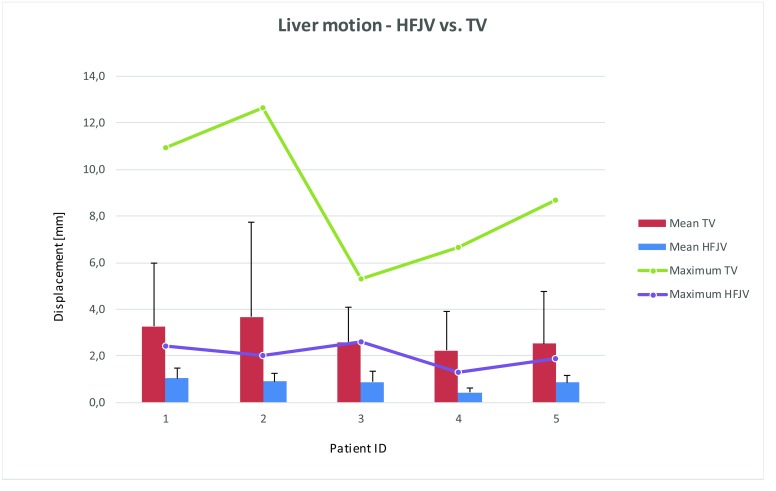
Liver displacement and lung ventilation. Displacement of measured point on liver surface during High-Frequency Jet Ventilation (HFJV) and standard ventilation (TV) Error bars mark standard deviation.

The mean Euclidean liver displacement was 0.80 (0.10 SD) mm and 2.90 (1.03 SD) mm for HFJV and TV respectively with maximum displacement going as far as 12 mm on standard ventilation (p=0.0001). Data shown in Figure 1.

Demographic data and ventilation readings metodological_study_1.xls Click here for additional data file. Copyright: © 2018 Galmén K et al. 2018 Data associated with the article are available under the terms of the Creative Commons Zero "No rights reserved" data waiver (CC0 1.0 Public domain dedication).

Liver positioning data 14-09-01-open-liver-all.xlsx Click here for additional data file. Copyright: © 2018 Galmén K et al. 2018 Data associated with the article are available under the terms of the Creative Commons Zero "No rights reserved" data waiver (CC0 1.0 Public domain dedication).

## Discussion

One of the most important challenges the anaesthesiologist faces perioperatively is the maintaining of the patient’s homeostasis and the facilitating of the course of surgery. In certain clinical situations, such as in stereotactic ablative procedures, it can be difficult to establish since there is a demand for keeping respiratory organ displacement to a minimum.

The recent investigation provides evidence for the claim that respiration induced liver motion during intervention can be reduced by more than two thirds when using HFJV instead of TV. This is the only study measuring this effect dynamically. This can have a decisive bearing on the risk of local recurrence rates and risk for collateral damage after image guided stereotactic treatment of liver tumours. The benefits in terms of radiation dose and respiratory organ shifting, when using HFJV in interventional radiology has previously been reported from several groups ^5, 6, 9^, but these were all conducted in the setting of a CT-guided ablation with non-dynamic measurements of target organ displacement.

Stereotactic navigation is often performed on rigid registration between the intraoperative target organ with the images obtained before the surgery. With this setting, soft tissue deformation and patient motion will affect the navigation system and can cause significant inaccuracy ^10^. The minimization of deformation-induced errors can be done in several ways. From experimental research point of view, the position of the moving target can be measured by implanted navigation aids or by using electromagnetic tracking devices ^10, 11^. Implantation of invasive needles is however not prudent in a clinical setting due to high risk of haemorrhage, tumour seeding, and long-term risks with leaving foreign bodies *in situ*.

Another approach is the mathematical modelling of mechanical tissue properties and organ motion in order to predict the target location based on a statistical model derived from preoperative 4D CT. This approach is frequently used in intensity modulated radiation therapies (IMRT) ^12^. The relationship between the respiratory cycle and the movement of a target is however complex to predict and not possible in real time due to highly intensive computational requirements and obvious risks of differences in outcome during the acquisition of preoperative images and a situation with artificial respiration and an open or laparoscopically affected abdomen.

Therefore, respiratory gating methods that reliably reproduce a known breathing stage (temporarily disconnecting endotracheal tube in anaesthetized patients) seem to be a more reliable approach ^12, 13^. An overall internal target movement of 1.41 ± 0.75 mm was reported. However, periods of apnea are usually limited to 1–2 minutes depending on the health condition of the patient. HFJV overcomes these restrictions.

Use of HFJV outside the ENT and Thorax suites have been the subject of several, but rather anecdotal reports. In minimally invasive oncological procedures HFJV have been used in percutaneous, laparoscopic as well as in open approaches ^3, 5, 6, 14^. In cardiology it has been beneficial in catheter ablations ^15^. In urology it can be helpful to minimize the numbers of shocks needed during ESWL-treatment ^15, 16^.

The present study is small and though the liver displacement data is solid, further studies on the physiological effects of HFJV is needed to elucidate the limitations. Carbon dioxide control is one of the important aspects of perioperative management. In the treatment protocol established during the study, it remained even more challenging because of the “less is better” strategy, favouring relatively low respiratory driving pressures.

Introducing HFJV in the management of computer-assisted abdominal surgery to a wider extent remains promising. The wider use of this method is, however, limited by the equipment availability and staff experience. Nevertheless, in the scale of a highly specialized centre, the acceptable skill level can easily be achieved, and the overall cost of the equipment as well as materials and utilities remains reasonable. HFJV is a promising lung ventilation modality for patients undergoing stereotactic surgical procedures in general anaesthesia when reduction of target organ displacement is crucial.

## Ethical considerations

All procedures performed were in accordance with the ethical standards of the institution at which the studies were conducted. Since this was a retrospective analysis of the clinical material collected before, the written consent has been obtained only from two patients still alive at the time when the decision of data analysis and publication have been made. Other three patients have already died.

## Data availability

The data referenced by this article are under copyright with the following copyright statement: Copyright: © 2018 Galmén K et al.

Data associated with the article are available under the terms of the Creative Commons Zero "No rights reserved" data waiver (CC0 1.0 Public domain dedication). 

Dataset 1: Demographic data and ventilation readings. metodological_study_1.xls 10.5256/f1000research.14873.d207212 ^17^

Dataset 2: Liver positioning data. 14-09-01-open-liver-all.xlsx 10.5256/f1000research.14873.d207213 ^18^

## References

[1] Zhang M Ma H Zhang J: Comparison of microwave ablation and hepatic resection for hepatocellular carcinoma: a meta-analysis. *Onco Targets Ther.* 2017;10:4829–39. 10.2147/OTT.S141968 29042794 PMC5633279

[2] Fukuhara T Aikata H Hyogo H: Efficacy of radiofrequency ablation for initial recurrent hepatocellular carcinoma after curative treatment: Comparison with primary cases. *Eur J Radiol.* 2015;84(8):1540–45. 10.1016/j.ejrad.2015.04.020 25979193

[3] Engstrand J Toporek G Harbut P: Stereotactic CT-Guided Percutaneous Microwave Ablation of Liver Tumors With the Use of High-Frequency Jet Ventilation: An Accuracy and Procedural Safety Study. *AJR Am J Roentgenol.* 2017;208(1):193–200. 10.2214/AJR.15.15803 27762601

[4] Galmén K Harbut P Freedman J: High frequency jet ventilation for motion management during ablation procedures, a narrative review. *Acta Anaesthesiol Scand.* 2017;61(9):1066–74. 10.1111/aas.12950 28804874

[5] Biro P Spahn DR Pfammatter T: High-frequency jet ventilation for minimizing breathing-related liver motion during percutaneous radiofrequency ablation of multiple hepatic tumours. *Br J Anaesth.* 2009;102(5):650–3. 10.1093/bja/aep051 19346232

[6] Abderhalden S Biro P Hechelhammer L: CT-guided navigation of percutaneous hepatic and renal radiofrequency ablation under high-frequency jet ventilation: feasibility study. *J Vasc Interv Radiol.* 2011;22(9):1275–8. 10.1016/j.jvir.2011.04.013 21703873

[7] Fritz P Kraus HJ Mühlnickel W: High-frequency jet ventilation for complete target immobilization and reduction of planning target volume in stereotactic high single-dose irradiation of stage I non-small cell lung cancer and lung metastases. *Int J Radiat Oncol Biol Phys.* 2010;78(1):136–42. 10.1016/j.ijrobp.2009.07.1678 19910142

[8] Engstrand J Nilsson H Jansson A: A multiple microwave ablation strategy in patients with initially unresectable colorectal cancer liver metastases - A safety and feasibility study of a new concept. *Eur J Surg Oncol.* 2014;40(11):1488–93. 10.1016/j.ejso.2014.05.003 24933395

[9] Denys A Lachenal Y Duran R: Use of high-frequency jet ventilation for percutaneous tumor ablation. *Cardiovasc Intervent Radiol.* 2014;37(1):140–6. 10.1007/s00270-013-0620-4 23636246

[10] Clifford MA Banovac F Levy E: Assessment of hepatic motion secondary to respiration for computer assisted interventions. *Comput Aided Surg.* 2002;7(5):291–9. 10.1002/igs.10049 12582982

[11] Maier-Hein L Müller SA Pianka F: Respiratory motion compensation for CT-guided interventions in the liver. *Comput Aided Surg.* 2008;13(3):125–38. 10.3109/10929080802091099 18432412

[12] Matney JE Parker BC Neck DW: Target localization accuracy in a respiratory phantom using BrainLab ExacTrac and 4DCT imaging. *J Appl Clin Med Phys.* 2011;12(2):3296. 10.1120/jacmp.v12i2.3296 21587171 PMC5718671

[13] Widmann G Schullian P Haidu M: Respiratory motion control for stereotactic and robotic liver interventions. *Int J Med Robot.* 2010;6(3):343–9. 10.1002/rcs.343 20632255

[14] Stillström D Nilsson H Jesse M: A new technique for minimally invasive irreversible electroporation of tumors in the head and body of the pancreas. *Surg Endosc.* 2017;31(4):1982–5. 10.1007/s00464-016-5173-6 27572065 PMC5346119

[15] Goode JS Jr Taylor RL Buffington CW: High-frequency jet ventilation: Utility in posterior left atrial catheter ablation. *Heart Rhythm.* 2006;3(1):13–9. 10.1016/j.hrthm.2005.09.013 16399046

[16] Cormack JR Hui R Olive D: Comparison of two ventilation techniques during general anesthesia for extracorporeal shock wave lithotripsy: high-frequency jet ventilation versus spontaneous ventilation with a laryngeal mask airway. *Urology.* 2007;70(1):7–10. 10.1016/j.urology.2007.03.045 17656197

[17] Galmén K Freedman J Toporek G: Dataset 1 in: Clinical application of high frequency jet ventilation in stereotactic liver ablations – a methodological study. *F1000Research.* 2018 10.5256/f1000research.14873.d207212 PMC6113879 30271582

[18] Galmén K Freedman J Toporek G: Dataset 2 in: Clinical application of high frequency jet ventilation in stereotactic liver ablations – a methodological study. *F1000Research.* 2018 10.5256/f1000research.14873.d207213 PMC6113879 30271582

