# Supplementary material for: Clinical application of high frequency jet ventilation in stereotactic liver ablations – a methodological study
Source: F1000Res. 2018 Sep 10;7:773. Originally published 2018 Jun 19. [Version 2] doi: 10.12688/f1000research.14873.2 (PMC6113879; doi:10.12688/f1000research.14873.2)
Supplement: Peer review history

# Peer review: Referee response for version 1

**DOI:** 10.5256/f1000research.16189.r37294

**Published:** 2018-08-28

**Authors:** Janusz Trzebicki, Jan Pluta

**Affiliations:** 1 First Department of Anesthesiology and Intensive Care, Medical University of Warsaw, Warsaw, Poland

The application of hight frequency jet ventilation (HFJV) during stereotactic thermalablation of liver tumours is an interesting method for minimising respiration related movements of this organ.

 The authors presented the results of a methodological study which includes five patients who underwent open computer-assisted liver thermalablation under general anesthesia using HFJV.

 The aim of this study was to compare HFJV with conventional ventilation (CV) in terms of influence of these techniques of ventilation on respiration related movements of liver.

 The liver movements range during both methods of ventilation were measured and compared in every patient. The applied measurement method allowed safe assessment of the dynamic movements of the organ.

 The results showed that HFJV in comparison to CV significantly reduced liver movements.

 The authors concluded that HFJV is a promising method of lung ventilation for patients qualified for surgery when reduction of target organ displacement is crucial.  

 Application of HFJV instead CV allowed better immobilisation of liver and therefore  it may allow us to perform ablation safely and more effectively. 

 So far, only a few  articles present this topic and the analysed groups of patients are limited.  That is why we need more well-designed studies evaluating HFJV during ablation procedures, such as the study written by Galmen *et al*.  In my opinion it is an important and correctly conducted study.

 The authors developed and described the new methodology of dynamic measurements of intraoperative liver movements related to lung ventilation. 

 This study provides new arguments for using HFJV for patients undergoing during stereotactic thermal ablation of liver tumours or others organs.

 Minor comments:

- On figure 1.

The term "standard ventilation” has two different  abbreviations in the article: on the chart SV abbreviation is used while in the description below we may find  TV.

- To include patient in the study  the number of metastases (as stayed in method section) should be  less than 20, whereas  one patient (table 1, ID-5) had 30.

We have read this submission. We believe that we have an appropriate level of expertise to confirm that it is of an acceptable scientific standard.

# Response

**Published:** 2018-08-30

**Authors:** Piotr Harbut

**Affiliations:** Department of Clinical Sciences, Department of Anesthesiology and Intensive Therapy, Danderyd University Hospital, Karolinska Institute, Stockholm, Sweden

We would like to thank professor Trzebicki for his positiv input. The suggested terminological changes in definition of conventional lung ventilation will be done in the manuscript soon. We choose the TV abbreviation for tidal volume ventilation.

 The second comment concerning the number of metastases in case 5 was the same as professor Sandströms and the appopriate explanation will be done in Discussion chapter.

# Peer review: Referee response for version 1

**DOI:** 10.5256/f1000research.16189.r36747

**Published:** 2018-08-06

**Authors:** Per Sandström

**Affiliations:** 1 Department of Surgery, Linköping University, Linköping, Sweden; 2 Department of clinical and experimental medicine, Linköping University, Linköping, Sweden

Galmén *et al *have written an important paper comparing liver movement during computer-assisted liver thermal ablation using high frequency jet-ventilation (HFJV) compared to conventional ventilation. The study includes 5 patients treated with open liver tumor ablations and both methods are used in each patient showing a significant and clinically relevant reduced liver movement with HFJV. 

 As pointed out by the authors, liver displacement must be as small as possible when performing liver ablations, to reduce the risk of missing the lesion. 

 A few minor comments

 In the method section it is stated that only patients with less then 20 tumors were included, but according to table 1. patient number 5 had 30 lesions, maybe this should be corrected.

 The reason for treating these patients with open surgery is unclear to me and this could possibly be explained in the method section.

 In the discussion the abbreviation ENT is not explained but may possibly be of value for some readers. 

 It would be most interesting to see the same kind of study performed in the percutaneous setting.

I have read this submission. I believe that I have an appropriate level of expertise to confirm that it is of an acceptable scientific standard.

# Response

**Published:** 2018-08-13

**Authors:** Piotr Harbut

**Affiliations:** Department of Clinical Sciences, Department of Anesthesiology and Intensive Therapy, Danderyd University Hospital, Karolinska Institute, Stockholm, Sweden

We thank professor Sandstrom for his comments and the opportunity to clearify some points.

1. One patient turned out to have more than 20 metastases at time of surgery, but not on the imageing where the treatment was allocated. Rather than to close up, a new evaluation was done during surgery using intraoperative ultrasound and it was found that ablations could be done leaving an adequate volume of functioning liver parenchyma, why this was the course taken.

2. Open surgery was used because at this time the hardware and software used for lesion tracking was only adapted for open surgery. We now use a laparoscopic approach instead and have done for a few years.

3. ENT is of course ear, nose and throat. This should have been spelled out in the text.

4. A dynamic study of liver motion with respiration could indeed be studied using flouroscopy or even better, tracking an electromagnetic intravascular probe introduced as far peripherally as possible in the liver. We have not done this but the suggestion is very good since this is an obvious problem during CT-guided interventions.
